# Failed Reverse Total Shoulder Arthroplasty Caused by Recurrent *Candida glabrata* Infection with Prior *Serratia marcescens* Coinfection

**DOI:** 10.1155/2014/142428

**Published:** 2014-11-06

**Authors:** John G. Skedros, Kendra E. Keenan, Wanda S. Updike, Marquam R. Oliver

**Affiliations:** ^1^The University of Utah Department of Orthopaedic Surgery, Salt Lake City, UT 84108, USA; ^2^Intermountain Medical Center, Salt Lake City, UT 84157, USA; ^3^Utah Orthopaedic Specialists, Salt Lake City, UT 84107, USA; ^4^Alpine Internal Medicine, Salt Lake City, UT 84102, USA; ^5^St. Marks Medical Center, Salt Lake City, UT 84124, USA; ^6^The University of Utah Department of Internal Medicine, Salt Lake City, UT 84132, USA

## Abstract

This report describes a 58-year-old insulin-dependent diabetic male patient who initially sustained a proximal humerus fracture from a fall. The fracture fixation failed and then was converted to a humeral hemiarthroplasty, which became infected with *Candida glabrata* and *Serratia marcescens*. After these infections were believed to be cured with antibacterial and antifungal treatments and two-stage irrigation and debridement, he underwent conversion to a reverse total shoulder arthroplasty. Unfortunately, the *C. glabrata* infection recurred and, nearly 1.5 years after implantation of the reverse total shoulder, he had a resection arthroplasty (removal of all implants and cement). His surgical and pharmacologic treatment concluded with (1) placement of a tobramycin-impregnated cement spacer also loaded with amphotericin B, with no plan for revision arthroplasty (i.e., the spacer was chronically retained), and (2) chronic use of daily oral fluconazole. We located only three reported cases of *Candida* species causing infection in shoulder arthroplasties (two *C. albicans*, one *C. parapsilosis*). To our knowledge, a total shoulder arthroplasty infected with *C. glabrata* has not been reported, nor has a case of a *C. glabrata* and *S. marcescens* periprosthetic coinfection in any joint. In addition, it is well known that *S. marcescens* infections are uncommon in periprosthetic joint infections.

## 1. Introduction

Although* Candida* infections following total joint arthroplasty have historically had a low prevalence in humans they are becoming more common [1–4]. Of all* Candida* infections, 11–16% are caused by* C. glabrata* but only 0.5 to 2% of prosthetic joints become infected with any pathogen [1, 5, 6]; coagulase-negative* Staphylococci* and* Staphylococcus aureus* account for >50% of cases [7]. Our review of the English literature of fungal-infected total joint arthroplasties revealed cases with these* Candida* species, in order from the most to the least common (with respect to all reported fungal organisms):* C. albicans *(~50–55% of cases),* C. parapsilosis* (~20–25% of cases),* C. glabrata* (~8% of cases),* C. tropicalis *and* C. pelliculosa* (<5% of cases), and* C. lipolytica, C. guilliermondii*,* C. famata*, and* C. lusitaniae* (only 1 case reported for each of these four species) [1, 2, 4–6, 8–20]. In this list of reports, we located fewer than 20 cases of* C. glabrata *following total joint arthroplasty and all of these occurred in hips and knees. In nearly half of these cases where* C. glabrata* was the cause, the outcome was poor, including resection arthroplasty, joint fusion, and amputation [2, 5, 13, 21]. Of these cases that we reviewed, the management of the infection typically involved some form of resection of prosthetic components with or without replacement and/or irrigation and debridement (I and D) and pharmacologic therapy with antifungal drugs. The management of these cases typically involved the two-stage revision approach recommended for bacterial-infected joint prostheses, which includes the use of a cement spacer in the first stage. More recently, the addition of antifungal drugs into a spacer during these revisions has been explored as well as single-stage revisions without the antifungal drugs in the spacer [22–24].

We located only three reported cases of* Candida *species causing infection in shoulder arthroplasties (two* C. albicans*, one* C. parapsilosis*) [10, 17, 18]. To our knowledge, a total shoulder arthroplasty infected with* C. glabrata* has not been reported. In addition, it is well known that* S. marcescens* infections are not common periprosthetic joint infections (PJI). Kuiper et al. (2013) [4] reported that bacteria were also cultured in one-third of the 164 patients that they described (~85% were* Candida* infected prosthetic hip and knee joints, and ~15% were non-*Candida* infected prosthetic hip and knee joints). Coagulase-negative* Staphylococcus *was also cultured in 26 patients, methicillin-sensitive* Staphylococcus aureus *(MSSA) in 13 patients, and methicillin-resistant* Staphylococcus aureus *(MRSA) in seven patients. To our knowledge, there have been no prior cases reported of a* C. glabrata *either alone in a shoulder arthroplasty or coinfected with* S. marcescens* in any prosthetic arthroplasty. We were able to locate one case of a fungal periprosthetic joint infection that had previously been infected with* S. marcescens*, but this was in a hip and the fungal isolate was* C. albicans* [22].

This report describes a patient who had a total shoulder arthroplasty with a recurrent* Candida glabrata *infection that was also previously coinfected with the bacteria* Serratia marcescens *(Gram-negative bacilli); these organisms rarely infect total joint arthroplasties [4, 25–27]. Our case is also unusual because the surgical and pharmacologic treatment concluded with (1) placement of a tobramycin-impregnated cement spacer also loaded with amphotericin B, with no plan for revision arthroplasty (i.e., the spacer was chronically retained), and (2) chronic use of daily oral fluconazole.

## 2. Case Report

**Figure 1:**
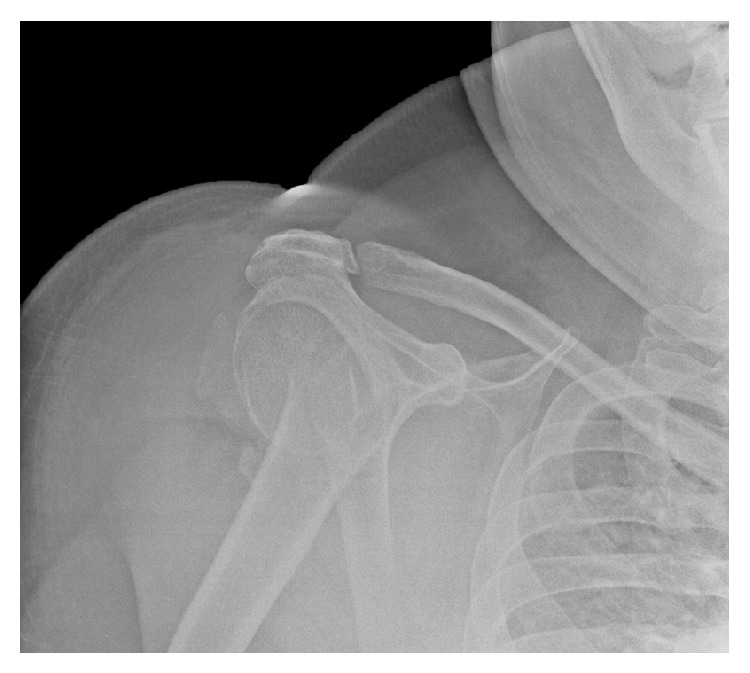
Radiograph of the patient's shoulder after the initial injury.

**Figure 2:**
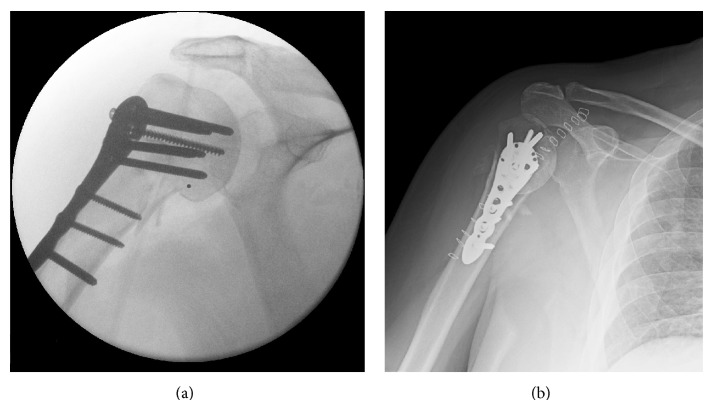
Radiograph showing the proximal humerus after reconstruction with a plate and screws (a) during surgery and (b) 14 days later when the screws had pulled out from the humeral head.

**Figure 3:**
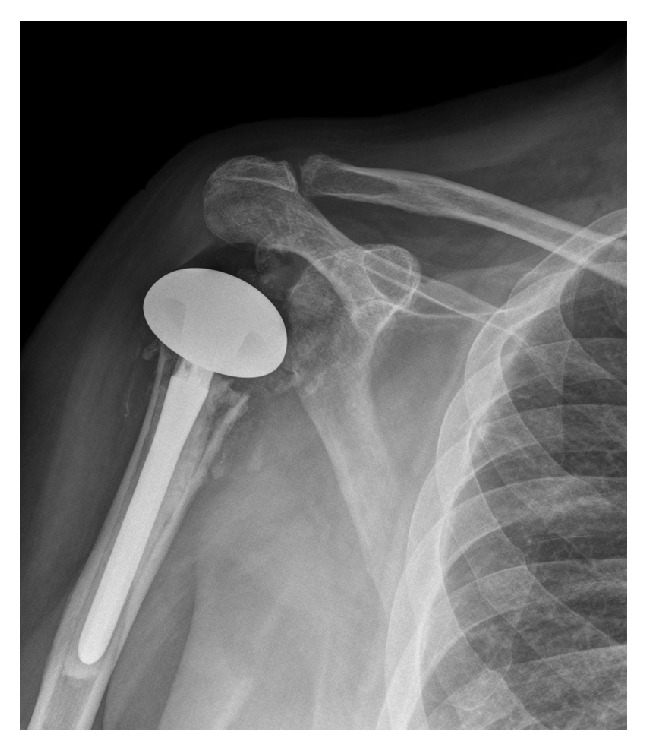
Radiograph showing the hemiarthroplasty.

**Figure 4:**
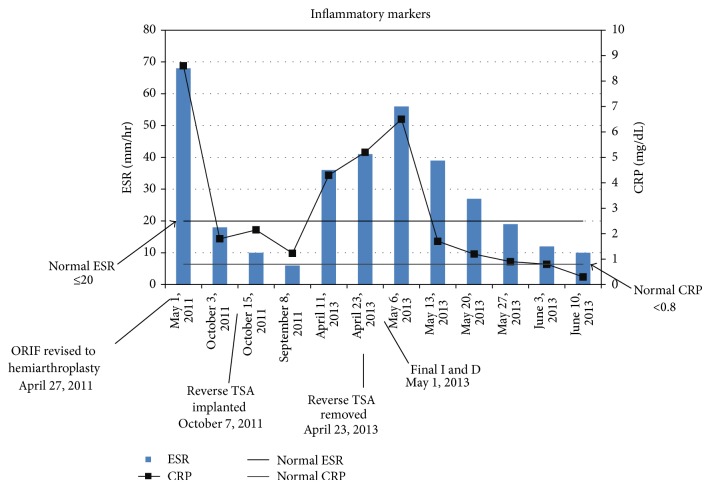
Inflammatory markers: C-reactive protein (CRP) and erythrocyte sedimentation rate (ESR) values throughout the patient's course of treatment. Note that the bars are not scaled accurately along the abscissa with respect to time.

An obese (BMI = 30.1) 58-year-old left-hand-dominant male presented to our clinic with a cement spacer that was placed two months before for the treatment of an infected hemiarthroplasty of the right shoulder. He had insulin-dependent diabetes, hypertension, sleep apnea, chronic obstructive pulmonary disease, and a history of two minor strokes (causing mild gait ataxia) and was in a chronic pain management program for disabling back pain. His right shoulder problems began five months before when he sustained a right proximal humerus three-part fracture after a ground level fall during a hypoglycemic attack (Figure 1). The fracture was treated with open reduction and internal fixation (ORIF) with a metal plate and locking screws (Figure 2(a)). Increased pain with unadvised shoulder motion three weeks later resulted in the screws pulling out from the humeral head (Figure 2(b)). This was revised to a cemented hemiarthroplasty (Figure 3). There were no unusual findings or complications associated with the revision. At that time no serum inflammatory markers had been obtained. All subsequent inflammatory markers are shown in Figure 4.

Although the referring surgeon did not initially suspect an infection, cultures taken at that hemiarthroplasty surgery grew* C. glabrata* and susceptibility testing was done (Table 1). An infectious disease specialist instituted intravenous (i.v.) caspofungin (50 mg i.v./day) for six weeks.

Five weeks later the mild erythema around the incision persisted and radiographic lucencies increased around the prosthesis. Eight cc of fluid aspirated from the joint grew* C. glabrata* and* S. marcescens*. Soon thereafter a draining fistula formed at the incision.

**Figure 5:**
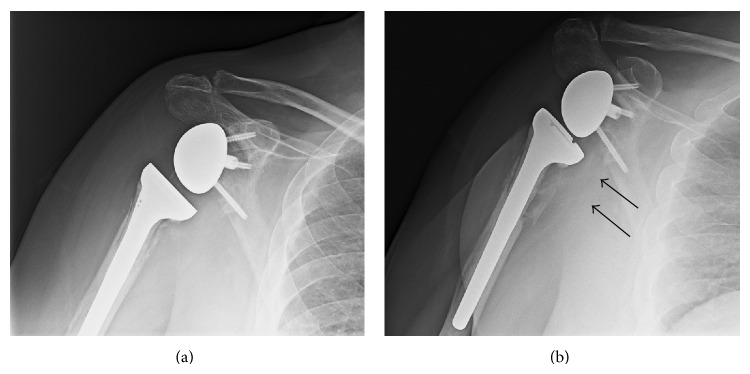
Radiograph showing (a) the reverse shoulder arthroplasty five months after implantation and (b) the reverse shoulder arthroplasty with the enlarged lucencies (arrows) at 18 months after implantation, which is just prior to its removal.

Resection arthroplasty (with complete cement removal) was done with placement of a handmade antibiotic-loaded (gentamicin augmented with vancomycin and tobramycin) cement spacer. Treatment also included piperacillin/tazobactam (4.5 grams i.v./8 hours) and micafungin (150 mg i.v./day) for six weeks, based on susceptibility results from cultures taken during revision to hemiarthroplasty five weeks before (Tables 1 and 2). Two months later he came to our clinic for the possibility of a reverse shoulder replacement. At that time aspiration of the right shoulder joint revealed scant fluid and no growth on cultures. Additional imaging studies (e.g., bone scan, gallium scan, or MRI scan) were not done. The spacer was removed, the joint was debrided, and a reverse total shoulder replacement was implanted (Figure 5(a)). There was no residual cement and no evidence of infection at the time of surgery, including no evidence of organisms or acute inflammation in frozen sections.

The patient showed good overall improvement in pain and function until one year later when he complained of pain and a sense of shoulder instability (but without dislocation) after falling on his right shoulder two months before. Radiographs showed lucencies around the proximal humerus in addition to increased sclerosis at the proximal-medial humerus. It was speculated that a nondisplaced proximal humerus fracture had occurred but did not result in loss of fixation of the humeral stem. The shoulder pain and function progressively improved.

Three months later (15 months after the reverse total shoulder) the patient complained of new-onset dull pain in his shoulder. Radiographs showed increased lucencies in the upper humerus. The lucencies increased over the following month (Figure 5(b)) and there were elevations in his C-reactive protein (CRP) (4.3 mg/dL; normal <0.8) and erythrocyte sedimentation rate (ESR; 36 mm/hr; normal ≤20) (Figure 4). An aspiration of fluid from the shoulder grew* C. glabrata.* Susceptibility testing was also done at this time and directed the pharmacologic management of the patient's infection (Table 1).

Irrigation and debridement (I and D) and resection arthroplasty were then performed. Cement beads were placed in the joint area and a pencil-shaped piece of cement was inserted into the medullary canal of the humerus. The cement included one packet (41 grams) that was manufactured with 1 gram of tobramycin, and a total of 500 mg of conventional amphotericin B powder (XGEN Pharmaceuticals Inc.) was also added before the addition of the monomer [28]. Treatment also included i.v. micafungin (150 mg/day) for eight weeks. Two weeks later a second I and D was performed and the cement was replaced with a handmade cement spacer that included two packets with tobramycin (1 gram/packet) and 500 mg of conventional amphotericin B powder.

Radiographs taken one month later showed the distal end of the spacer protruding through the distal aspect of the humerus fracture that had occurred during the I and D. This was not surgically corrected because the discomfort there subsided. The ESR and CRP were also within normal limits by one month after the final I and D (Figure 4) and remained normal on subsequent visits. An infectious disease specialist and the patient's medical physician recommended avoiding future shoulder arthroplasty because of risk of relapse. After the treatment with micafungin, we then opted for chronic suppressive oral fluconazole (400 mg/day) due to the high likelihood that this infection was not cured and his chronically immunocompromised state. Chronic use of oral fluconazole for prophylaxis for fungal infection is not known to be associated with significant side effects [29–31].

**Figure 6:**
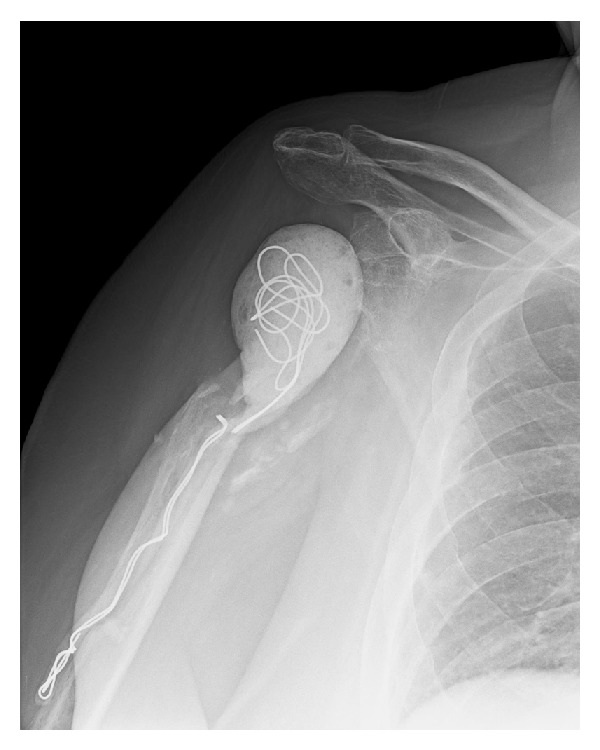
Radiograph at one year after the final I and D showing the cement spacer that the patient was asked to “live with.”

**Figure 7:**
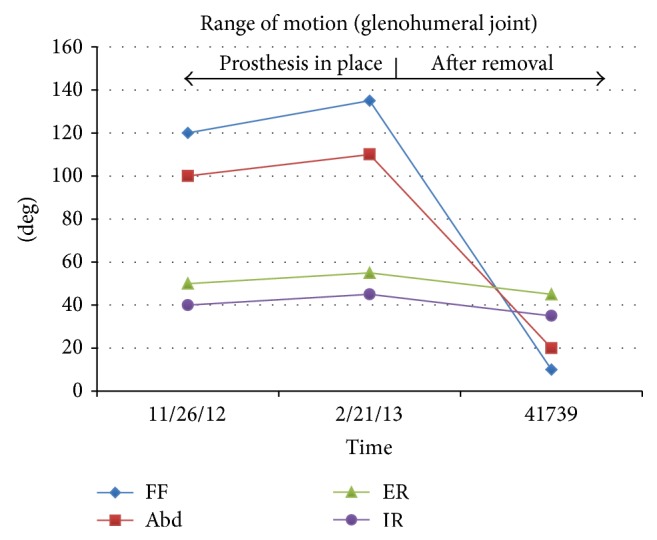
Range of motion (ROM) values before removal of the reverse prosthesis and one year after the cement spacer was placed. Labels are forward flexion (FF), abduction (Abd), external rotation (ER), and internal rotation (IR).

At one year after placement of the final cement spacer (Figure 6) there was no evidence of infection and his shoulder pain was moderate with attempted shoulder use. He was taking regular pain medications (hydrocodone) primarily for chronic low back pain and antispasmodic medications (cyclobenzaprine) for shoulder discomfort. Outcome data in Table 3 and range of motion values in Figure 7 showed very poor shoulder function. Although his shoulder function remained poor at 1.5 years after placement of the final cement spacer, a decision was agreed upon by all of the consulting physicians to continue oral suppressive therapy and take no further surgical actions due to his risk of recurrence and immunocompromised state.

## 3. Discussion

This is a unique case of a diabetic man with multiple comorbidities who had coinfection with yeast (*Candida glabrata*) and bacteria (*Serratia marcescens*) subsequent to a failed ORIF and revision to a humeral hemiarthroplasty. Fungal infections following orthopaedic surgeries are uncommon and the bacterial coinfection seen in this patient is even less common. Furthermore, the* C. glabrata* infection recurred despite all evidence suggesting it was eradicated. Treatment ultimately included resection arthroplasty, retention of the cement spacer, i.v. micafungin for eight weeks, and chronic oral antifungal therapy.

We successfully eradicated the* S. marcescens *infection with a six-week course of i.v. piperacillin/tazobactam in addition to a cement spacer loaded with antibiotics to which this organism was sensitive (Table 2). However, if the* S. marcescens* infection had been a multiple-drug resistant strain, then high dose meropenem may have been needed [32]. But this was not the case for our patient. The surgical treatment of PJI caused by* S. marcescens* is the same as treatment of other Gram-negative bacilli bacterial infections in a prosthetic joint, which involves a two-stage revision [26, 32, 33].

The cause of our patient's initial infection is unknown. For example, he did not have traditional risk factors such as fungemia,* Candida* infection, central line infection, or prolonged antibiotic use. Common causes for* C. glabrata *and* S. marcescens *include hematogenous spread, prior colonization of the pathogens, and/or introduction of the pathogens at the surgical site [16, 26, 34]. Patients at high risk for these types of postsurgical infections also include i.v. drug users and, as in our patient, those with reduced immunity and/or extensive comorbidities, especially diabetes [3, 21, 35].

The recurrence of the* C. glabrata* infection in our patient appears to be consistent with the high recurrence rate (12–50%) of* Candida *infections in total hip and knee arthroplasties [4, 36, 37]. The lack of well-developed guidelines for treatment of patients with fungal and bacterial coinfections and for recurrent* C. glabrata* infected joint arthroplasties [4, 38] prompted treatment consistent with that of a complicated bacterial infection [16, 21]. As shown by the literature review of Kuiper et al. [4], appropriate antifungal therapy (i.v. and also in the spacer) and a two-stage revision can result in an 85% success rate (67/79 patients) [4], which approaches the success rate of staged revisions for bacteria infected hip and knee arthroplasties (87–91%) [7, 39, 40]. By contrast, Kuiper et al. also tabulated these less favorable cure rates for fungal-infected hip and knee arthroplasties: (1) 4/22 (18%) with debridement, antifungal antibiotics, irrigation, and prosthesis retention; (2) antifungal therapy alone (0/3; 0%); and (3) revision at time of first resection/debridement (1/2; 50%). In a subset of 119 patients that they reviewed, 14 add permanent resection arthroplasty. Finally, of 79 joints that were reimplanted, 62 were cured, 5 were cured after “additional debridement,” and 12 failed this treatment.

For fungal-infected total joint arthroplasties, the Infectious Diseases Society of America recommends removal of the arthroplasty in most patients, with organism-specific i.v. therapy for at least six weeks with subsequent reimplantation [41]. If removal is not an option, for instance due to the patient's poor health, chronic suppression with an oral antifungal is recommended [42].

We placed a single spacer and performed a single I and D in accordance with the literature that was available at the time for treating similar bacterial PJI. However, since that time additional literature has become available specifically dealing with fungal-infected joints. In view of this new literature and the difficulty in curing these infections, we propose that an additional I and D and spacer would have been helpful in the treatment of our patient. However, Klatte and coworkers [22, 43] have recently proposed that a single-stage revision may be effective in the treatment of fungal-infected knees and hips with and without previous bacterial coinfections and also in bacterial PJI of the shoulder because of the reduction in the number of operations. But due to the rarity of these infections these were small studies and they included relatively more common yeast and bacteria than what our patient had. In addition, they excluded four of their total 14 patients with prior fungal PJI (two died, and one recurred within two weeks, and one with an acetabular abnormality). Consequently, the effectiveness of one-stage revision might be overstated in their study. It is conceivable that these four excluded patients could have had recurrent infection and, had they been included in the study, the failure rate could be much higher than the 10% that they report. Furthermore, two-stage I and Ds are recommended for* Serratia marcescens *infections, like the one that our patient had, which is also the same recommendation for Gram-positive PJI [33]. This supports our decision for using the two-stage approach. Nonetheless, further investigation is needed to determine the best course of action in terms of treatment of patients with rare coinfections involving a fungal organism and especially for patients who are chronically immunocompromised.

In addition to the limited guidelines we had available to guide us in our patient's treatment, our reasons for not placing a second antibiotic-loaded spacer and performing a second I and D before implanting the reverse shoulder included (1) no evidence from preoperative joint fluid aspiration and intraoperative frozen sections of a recurrent infection and (2) the referring surgeon's operative note stating that an antifungal drug was added to the spacer (prior to the reverse shoulder). However, it was retrospectively recognized that this was an error—subsequent personal communication with the referring surgeon revealed that the first spacer only contained an antibacterial agent.

When it was clear that the* C. glabrata* infection had recurred, a more aggressive approach was taken to eradicate the infection, including two I and Ds with conventional amphotericin B in the cement beads and spacer. Wu and Hsu [23] reported successful treatment of an initial (not recurrent)* C. albicans* infection in the setting of a revision total knee arthroplasty. They used cement with one gram of conventional amphotericin B. Their report and that of Graw et al. [24] were the basis for our addition of amphotericin B in the cement used in our I and D procedures. However, we used 500 mg of amphotericin B because the one gram dose per batch of cement used by Wu and Hsu [23] greatly exceeded the 50 mg/batch advocated by Graw et al. Consequently, nephrotoxicity was our concern. However, we later became aware of a laboratory study showing minimal, clinically nonsignificant elution of amphotericin B from a cement spacer loaded with 200 mg of this drug [28]. This potentially is due to its hydrophobicity and propensity to form micelles [44]. While this lends support to the use of higher doses, the safety and effectiveness of “higher” doses have not been established. Liposomal amphotericin B might become the better choice because of its probable enhanced elution [38], and sophisticated porosity/pharmacologic modifications of bone cement spacers are also being considered [44, 45]. By contrast, Klatte et al. [22] elected not to use antifungal drugs in their spacers due to their poor elution from the cement. But, similar to the discussion with regard to their study above, the surgical and pharmacological strategies that they employed might be suboptimal in view of the guidelines recommended by the Infectious Disease Society of America [41]. Sealy et al. [46] suggest that most antifungal agents are not suitable for use in cement spacers for the treatment of deep fungal infections because of their ineffective elution. However, Miller et al. [47] showed that voriconazole has increased elution from bone cement as dose increases, but compressive strength of the cement progressively decreases with elution.

The review of Kuiper et al. [4] is potentially useful for future studies that examine elution dynamics of various concentrations of antifungal agents in porosity-modified cement spacers. Of the cement spacers used in 86 fungal-infected hip and knee arthroplasties that they reviewed, sixty-eight were loaded with antibacterial antibiotic agents, five with antifungal agents, and seven with both. The exact doses of antifungal agents were mentioned by seven authors: amphotericin B in nine patients (between 187.5 mg and 1,200 mg per cement batch (40 g)), amphotericin B and voriconazole in one patient (250 mg and 1,000 mg per batch, resp.), fluconazole in one patient (200 mg in a spacer), and itraconazole in one patient (250 mg in a spacer). In two patients, fluconazole-loaded bone cement beads were implanted (2,000 mg per batch).

Failure to use an antifungal agent in the initial cement spacer (prior to the reverse shoulder prosthesis) and our decision not to perform one more I and D with placement of a second antifungal-loaded spacer for at least six weeks before implanting the reverse total shoulder arthroplasty, coupled with the persistent nature of* C. glabrata*, likely contributed to the recurrent infection. For future cases that resemble our very unhealthy patient and the circumstances and sequelae of his fracture treatment, we suggest that after the index yeast infection occurs it might be reasonable to employ a three-stage approach, with two I and D procedures six or more weeks apart and each with the placement of an antifungal-loaded spacer. However, we suggest this only because the previous antifungal treatment for our patient's initial and recurrent* C. glabrata* infections was inadequate. Aspiration culture and frozen section observations may have low sensitivity in* Candida* infection after systemic antifungal treatment [48]. Repeated aspiration culture and inflammatory markers should therefore be considered before reimplantation or another debridement surgery [49]. Recently improved cement spacers have become available for the treatment of infected total shoulder arthroplasties [50], and there are antifungal agents (as above) that are better than amphotericin B for impregnating into cement spacers. The determination of what agents to use should be made based on the most recent literature on the elution and efficacy of antifungal drugs in bone cement. Longer duration of i.v. antifungal treatment and use of a spacer with enhanced elution capacity might also be warranted. Additional research is needed to further develop treatment guidelines.

## Figures and Tables

**Table 1:** Susceptibility results of our patient's *C. glabrata* isolates to various antifungal drugs at the time of revision from the ORIF to the hemiarthroplasty and one week prior to the resection arthroplasty of the reverse total shoulder arthroplasty (RTSA).

Drug	3 weeks after injury (revision to hemiarthroplasty)	19 months after RTSA (1 week prior to removal)
Fluconazole^*^	8	4
Micafungin	≤0.008	≤0.008
Caspofungin	0.06	0.12
Voriconazole^*^	0.12	0.12
5-Fluorocytosine	≤0.06	≤0.06
Anidulafungin	≤0.03	≤0.016
Itraconazole^*^	0.5	0.25
Posaconazole	1	0.5
Amphotericin B^†^	≤0.5	≤0.5

^*^For fluconazole, itraconazole, and voriconazole, the susceptibility is dose dependent where the maximum possible level must be achieved (here the level for fluconazole is <32).

^†^For amphotericin B, an MIC >1 *μ*g/mL is considered resistant.

**Table 2:** Susceptibility results of our patient's *S. marcescens *isolate to various antibacterial drugs. The bolded portions of the table indicate the drugs that were used in our patient's treatment of *S. marcescens*. Gentamicin and tobramycin were only used in the cement spacer; vancomycin was not tested.

Drug	MIC
Amikacin^*^	<16
Amoxicillin/K clavulanate	>16/18
Ampicillin/sulbactam	>16/18
Ampicillin	>16
Aztreonam^*^	≤8
Cefazolin	>16
Cefepime^*^	≤8
Cefotaxime^*^	≤2
Cefotetan	≤16
Cefoxitin	16
Ceftazidime^*^	≤1
Ceftriaxone^*^	≤8
Cefuroxime	>16
Ciprofloxacin^*^	<1
Ertapenem^*^	≤2
**Gentamicin** ^*^	≤4
Imipenem^*^	≤4
Levofloxacin^*^	≤2
Meropenem^*^	≤4
Moxifloxacin^*^	≤2
**Piperacillin/tazobactam** ^*^	≤16
Tetracycline^†^	8
**Ticarcillin/K clavulanate** ^*^	≤16
**Tobramycin** ^*^	≤4
Trimethoprim/sulfamethoxazole^*^	≤2/38

^*^Susceptible.

^†^Intermediate.

No superscript = resistant; K = potassium.

**Table 3:** The patient's values for the DASH score [51, 52], WORC score [53], Simple Shoulder Test (SST) [54], and SF-36 [55] prior to the removal of the reverse shoulder prosthesis and at one year after the final spacer had been placed. For the SF-36 all questions are scored from 0 to 100 representing the highest level of functioning possible.

Outcome measure	Pre-op	Final outcome
DASH (best = 0)	58	90
WORC (best = 100%)	44	21
SST (best = 12 yes responses)	4	0
SF-36 (best = 100)	20	15
